# Association of fear of COVID-19 and resilience with psychological distress among health care workers in hospitals responding to COVID-19: analysis of a cross-sectional study

**DOI:** 10.3389/fpsyt.2023.1150374

**Published:** 2023-04-27

**Authors:** Haruhiko Midorikawa, Hirokazu Tachikawa, Natsuho Kushibiki, Keiko Wataya, Sho Takahashi, Yuki Shiratori, Kiyotaka Nemoto, Shinichiro Sasahara, Shotaro Doki, Daisuke Hori, Ichiyo Matsuzaki, Testuaki Arai, Kunihiro Yamagata

**Affiliations:** ^1^Department of Psychiatry, University of Tsukuba Hospital, Tsukuba, Japan; ^2^Department of Disaster and Community Psychiatry, Division of Clinical Medicine, Institute of Medicine, University of Tsukuba, Tsukuba, Japan; ^3^Majors of Clinical Sciences, Graduate School of Comprehensive Human Sciences, University of Tsukuba, Tsukuba, Japan; ^4^Department of Psychiatry, Division of Clinical Medicine, Institute of Medicine, University of Tsukuba, Tsukuba, Japan; ^5^Occupational and Aerospace Psychiatry Group, Division of Biomedical Science, Institute of Medicine, University of Tsukuba, Tsukuba, Japan; ^6^Department of Nephrology, Division of Clinical Medicine, Institute of Medicine, University of Tsukuba, Tsukuba, Japan

**Keywords:** COVID-19, mental health, fear, resilience, healthcare workers (HCWs)

## Abstract

**Background:**

It remains unclear how fear of COVID-19 and resilience are related to psychological distress based on occupations among healthcare workers (HCWs) in hospitals treating patients with COVID-19. We conducted a survey on the mental health of HCWs during the COVID-19 pandemic to determine the relationship between factors such as fear of COVID-19 and resilience as well as mental distress in each occupation of HCWs.

**Methods:**

We conducted a web-based survey among HCWs at seven hospitals treating COVID-19 patients in Japan from December 24, 2020 to March 31, 2021. A total of 634 participants were analyzed, and information regarding their socio-demographic characteristics and employment status was collected. Several psychometric measures were used, including the Kessler’s Psychological Distress Scale (K6), the fear of COVID-19 Scale (FCV-19S), and the Resilience Scale (RS14). Factors related to psychological distress were identified by logistic regression analysis. The association between job title and psychological scales was examined by one-way ANOVA, and *t*-tests were conducted to examine the association between the FCV-19S and hospital initiatives.

**Results:**

It was found that nurses and clerical workers were associated with psychological distress without considering FCV-19S or RS14; in a model that included FCV-19S, FCV-19S was associated with psychological distress, but job title was not; when RS14 was considered, resilience was protective. In terms of occupation, FCV-19S was lower among physicians and higher among nurses and clerical workers, while RS14 was higher among physicians and lower among other occupations. Having access to in-hospital consultation regarding infection control as well as to psychological and emotional support was associated with lower FCV-19S.

**Conclusion:**

Based on our findings, we can conclude that the level of mental distress differed by occupation and the differences in the fear of COVID-19 and resilience were important factors. In order to provide mental healthcare for HCWs during a pandemic, it is important to create consultation services that enable employees to discuss their concerns. In addition, it is important to take steps to strengthen the resilience of HCWs in preparation for future disasters.

## 1. Introduction

During the COVID-19 pandemic, the mental health of people worldwide deteriorated significantly, especially among healthcare workers (HCWs), who reported worse mental health than non-HCWs (1). This is believed to be due to factors specific to HCWs, such as physical and emotional exhaustion from treating COVID-19 patients, risk of infection, and fear of secondary transmission to family members, as well as discrimination and prejudice (2, 3). Based on a meta-analysis examining the psychological impact of COVID-19 on HCWs, the pooled prevalence of anxiety, depression, stress, post-traumatic stress syndrome, insomnia, psychological distress, and burnout was 34.4, 31.8, 40.3, 11.4, 27.8, 46.1, and 37.4%, respectively (4). The mental health of HCWs during the COVID-19 pandemic has also emerged as a major issue in Japan. For example, in a survey conducted among HCWs, 10% developed moderate-to-severe anxiety disorder and 27.9% developed depression (5). Additionally, an online cross-sectional survey of HCWs at a tertiary hospital revealed that 22.6% of the participants met the burnout criteria based on the Maslach Burnout Inventory–General Survey (6). Another study found that the prevalence rates of severe general and event-related distress worsened over time, and 8.6% of the hospital workers experienced suicide-related ideation in 2021 (7). Moreover, HCWs have been reported to have deteriorating mental health during outbreaks of infectious diseases, such as Severe Acute Respiratory Syndrome (SARS) and Middle East Respiratory Syndrome (MERS) (8). Given the long-term impact of the pandemic on mental health (9), as well as the possibility of emergence of mutant strains and new infections in the future, the impact of the COVID-19 pandemic on the mental health of HCWs is still an important issue 4 years after the outbreak began.

Several reports have been published on the factors related to the mental health of HCWs during the COVID-19 pandemic. In terms of gender, a higher prevalence of anxiety and depression was reported in females than in males (4), and in terms of age, mental health was worse among younger people (10). Associations of various factors, such as marital status, cohabitation, social support, employment status, and job description, have also been reported (11–13). In terms of occupations, nurses and other professionals reported more mental health problems than doctors (4, 14). Several studies in Japan have examined factors related to the mental health of HCWs during the COVID-19 pandemic from multiple perspectives. For instance, a cross-sectional survey of HCWs found that psychological distress was associated with occupations such as nurses, allied health professionals, and office workers/engineers; moreover, moral distress was not associated with psychological distress, but low resilience was (15). Other surveys conducted among HCWs showed that older and more resilient HCWs were less likely to develop depressive symptoms, and women, non-physicians, those who lived alone, and younger respondents had significantly greater psychological distress than their counterparts (5, 16). Moreover, nurses had the highest rates of depression, and younger and newer employees demonstrated the highest rates of depression independent of occupation (17). For HCWs at a national medical institution designated for COVID-19 treatment, chronic physical conditions were significantly associated with depressive symptoms (18). In another survey, frontline workers had increased odds of COVID-19-related discrimination, which was associated with PTSD symptoms and psychological distress, compared with second-line workers (19). According to a multi-center collaborative survey, COVID-19-related discrimination was significantly associated with subsequent depression and suicidal ideation among HCWs (20). However, the reasons for the differences in mental health among occupations in HCWs during the COVID-19 pandemic remain unclear. In particular, it is unclear whether fear of COVID-19, a factor unique to the COVID-19 pandemic, and resilience, an important concept as a protective factor for mental health, are associated with differences in mental health among occupations in HCWs. Studies showing the importance of stress coping skills, such as resilience (21, 22), defense mechanisms (23, 24), and personality traits, such as grit (25), highlight the need to consider not only sociodemographic characteristics but also psychological factors, such as fear of COVID-19 and resilience, for identifying factors related to the mental health of HCWs.

Therefore, we hypothesized that the reason behind the differences in mental health during the COVID-19 pandemic among HCWs in different occupations is not only because of differences in their sociodemographic characteristics, but also those in psychological factors such as fear of COVID-19 and resilience. Thus, we conducted a survey on the mental health of HCWs during the COVID-19 pandemic to determine the relationship between factors such as fear of COVID-19 and resilience as well as mental distress in each occupation of HCWs.

## 2. Materials and methods

### 2.1. Study design and participants

Data from an online questionnaire survey of seven hospitals in Ibaraki, Japan, treating patients with COVID-19 were analyzed in this cross-sectional study. An overview of the survey was widely announced at each hospital and participation was voluntary. Participants who gave informed consent on the web provided information regarding their socio-demographic characteristics and mental health. They were informed that they could discontinue their participation at any time. The survey period was from December 24, 2020 to March 31, 2021, and of the 709 respondents who completed the questionnaire, 634 (89.4%) with no missing values were included in the analysis.

### 2.2. Measures

We collected the following characteristics of the participants: facility affiliation (public/private hospital), gender (male/female), age group (20s/30s/40s/50s +), cohabitant (no/yes), occupation (Doctor/nurse or nursing assistant/pharmacist, laboratory technician, physical therapist, speech therapist or occupational therapist/clerical staff or other), workplace (ward/outpatient/other), and employment status (full-time/part-time), night shifts (no/yes), and COVID-19 related work (no/past/current). We also measured the Kessler’s Psychological Distress Scale (K6), the fear of COVID-19 Scale (FCV-19S), and the Resilience Scale (RS14) as indicators of mental health. Additionally, we inquired about the participants’ perceptions regarding the following four hospital initiatives: (1) training on infection control, (2) adequate supply of personal protective equipment, (3) availability for consultation regarding infection control at the hospital, and (4) availability of psychological and emotional support services at the hospital.

The K6 is a self-administered psychological scale with six items that are measured on a five-point Likert scale ranging from 0 to 4 points. The total score of K6 ranges from 0 to 24. This scale was developed to screen for mood and anxiety disorders (26). There is evidence of validity and reliability that supports the use of K6 in the Japanese population (27). A previous study (28) shows that K6 ≥ 5 points was adopted as the cut-off value to determine whether the participants were in moderate or higher psychological distress. Cronbach’s alpha for K6 was 0.894, indicating satisfactory reliability of the scale in the current study.

The FCV-19S is a seven-item self-administered psychological scale that uses a five-point Likert scale ranging from 1 to 5 points. The total score of FCV-19S ranges from 7 to 35 points. This scale was developed to measure the fear of COVID-19 (29). There is evidence of validity and reliability that supports the use of FCV-19S in the Japanese population (30, 31). Cronbach’s alpha for FCV-19S was 0.836, indicating satisfactory reliability of the scale in the current study.

The RS14 is a 14-item psychological scale that uses a seven-point Likert scale ranging from 1 to 7 points. The total score of the RS14 ranges from 14 to 98. This scale was developed to measure resilience, which is defined as a personality characteristic that moderates the negative effects of stress and promotes adaptation in response to it (32). There is evidence of validity and reliability that supports the use of RS14 in the Japanese population (33). Cronbach’s alpha for RS14 was 0.929, indicating satisfactory reliability of the scale in the current study.

### 2.3. Statistical analysis

First, we present the distribution of each variable in the groups with or without psychological distress. Second, binomial logistic regression analysis with psychological distress as the dependent variable was performed for models excluding RS14 and FCV-19S, including RS14, including FCV-19S, and including both RS14 and FCV-19S. Third, the Hosmer-Lemeshow test was used to evaluate the goodness of fit of the models. Fourth, for each model, the Variance Inflation Factor (VIF) of all the variables was assessed for multicollinearity. Fifth, a one-way analysis of variance was performed using the Bonferroni correction for multiple comparisons to compare the FCV-19S/RS14 for each job category. Finally, a *t*-test was performed for the association between FCV-19S and perceptions of hospital initiatives, and Cohen’s d was calculated as the effect size. A *p*-value of less than 0.05 is considered statistically significant. Additionally, all statistical analyses were performed using IBM SPSS Statistics (version 28, Armonk, NY, USA, 2021).

## 3. Results

The demographics of the participants are shown in Table 1. The majority of participants were female (*n* = 472) and in their 40s (*n* = 203). There were 492 participants who lived with another individual. In addition, 93 were doctors, 302 were nurses, 98 were pharmacists, laboratory technicians, physical therapists, occupational therapists, or speech therapists, and 141 were clerical workers and others. The majority of workplaces were hospital wards (*n* = 305), 585 were full-time, and 329 had night shifts; 65 were currently engaged in COVID-19-related activities. The psychological scales (mean ± standard deviation) of the participants were K6 6.0 ± 5.1, FCV-19S 18.9 ± 4.8, and RS14 61.8 ± 14.1.

**TABLE 1:** Characteristics of participants.

Variable		K6 < 5 *N* = 305		K6 ≥ 5 *N* = 329	
		**N**	**%**	**N**	**%**
Facility affiliation	Public	147	48.2	135	41.0
	Private	158	51.8	194	59.0
Gender	Male	97	31.8	65	19.8
	Female	208	68.2	264	80.2
Age group	20s	50	16.4	87	26.4
	30s	87	28.5	77	23.4
	40s	91	29.8	112	34.0
	50s +	77	25.2	53	16.1
Cohabitant	No	56	18.4	86	26.1
	Yes	249	81.6	243	73.9
Occupation	Doctor	65	21.3	28	8.5
	Nurse/nursing assistant	129	42.2	173	52.6
	Pharmacist laboratory technician PT/ST/OT	54	17.7	44	13.4
	Clerk/other	57	18.7	84	25.5
Work place	Ward	142	46.6	163	49.5
	Outpatient	45	14.8	56	17.0
	Other	118	38.7	110	33.4
Employment status	Full-time	281	92.1	304	92.4
	Part-time	24	7.9	25	7.6
Night shift	No	158	51.8	147	44.7
	Yes	147	48.2	182	55.3
COVID-19 related work	No/past	272	89.2	297	90.3
	Current	33	10.8	32	9.7

According to our logistic regression analysis, the model excluding FCV-19S/RS14 (Hosmer-Lemeshow test *p* = 0.94) showed that nursing (OR = 2.27, 95%CI 1.29–4.01), office/other (OR = 3.98, 95%CI 2.09–7.58), and night shifts (OR = 1.51, 95%CI 1.01–2.27) were associated with psychological distress (Model 1, Table 2). However, in the model including RS14 (Hosmer-Lemeshow test *p* = 0.83), RS14 (OR = 0.93, 95%CI 0.92–0.95) and clerical worker (OR = 2.94, 95%CI 1.46–5.94), but not nurses (OR = 0.96, 95%CI 0.51–1.81) and night shift (OR = 1.32, 95%CI 0.85–2.05) were associated with psychological distress (Model 2, Table 2). In the model including FCV-19S (Hosmer-Lemeshow test *p* = 0.65), FCV-19S (OR = 1.28, 95%CI 1.22–1.34) and living with another individual (OR = 0.56, 95%CI 0.35–0.89), were associated with psychological distress, but not job title and night shift (Model 3, Table 3). In the model with both RS14 and FCV-19S (Hosmer-Lemeshow test *p* = 0.11), RS14 (OR = 0.93, 95%CI 0.92–0.95), FCV-19S (OR = 1.29, 95%CI 1.22–1.36), and living with another individual (OR = 0.50, 95%CI 0.30–0.82) were associated, but not job title (Model 4, Table 3). The VIF was < 4 for all models, and no serious multicollinearity issues were observed.

**TABLE 2:** Logistic regression analysis of psychological distress (Model 1 and Model 2).

	Model 1				Model 2			
	**P**	**OR**	**95% CI**		**P**	**OR**	**95% CI**	
**Facility affiliation (ref. public)**								
Private	0.098	1.330	0.949	1.865	0.101	1.359	0.942	1.962
**Gender (ref. male)**								
Female	0.186	1.339	0.869	2.064	0.273	1.301	0.813	2.083
**Age group (ref. 40s)**								
20s	0.353	1.259	0.774	2.047	0.958	0.986	0.585	1.661
30s	0.132	0.716	0.463	1.106	0.139	0.698	0.433	1.124
50s +	0.221	0.743	0.462	1.195	0.991	1.003	0.599	1.678
**Cohabitant (ref. no)**								
Yes	0.182	0.752	0.494	1.143	0.093	0.680	0.434	1.067
**Occupation (ref. doctor)**								
Nurse/nursing assistant	**0.005**	**2.272**	**1.286**	**4.014**	0.156	1.565	0.843	2.903
Pharmacist laboratory technician PT/ST/OT	0.094	1.728	0.910	3.281	0.405	1.344	0.670	2.699
Clerk/other	**0.000**	**3.981**	**2.090**	**7.584**	**0.003**	**2.942**	**1.458**	**5.937**
**Workplace (ref. ward)**								
Outpatient	0.824	1.062	0.627	1.798	0.715	1.110	0.634	1.944
Other	0.192	0.756	0.496	1.151	0.227	0.756	0.480	1.191
**Employment status (ref. full-time)**								
Part-time	0.937	1.026	0.548	1.920	0.413	1.333	0.670	2.654
**Night shift (ref. no)**								
Yes	**0.045**	**1.512**	**1.009**	**2.265**	0.213	1.322	0.852	2.051
**COVID-19 related work (ref. no/past)**								
Current	0.248	0.718	0.409	1.259	0.683	0.878	0.469	1.642
**Fear of COVID-19 and resilience**								
FCV-19S								
RS14					**0.000**	**0.932**	**0.917**	**0.947**

The bold values indicate *p* < 0.05.

**TABLE 3:** Logistic regression analysis of psychological distress (Model 3 and Model 4).

	Model 3				Model 4			
	**P**	**OR**	**95% CI**		**p**	**OR**	**95% CI**	
**Facility affiliation (ref. public)**								
Private	0.352	1.195	0.821	1.740	0.451	1.169	0.779	1.753
**Gender (ref. male)**								
Female	0.376	1.242	0.769	2.006	0.398	1.247	0.748	2.080
**Age group (ref. 40s)**								
20s	0.337	1.302	0.760	2.231	0.933	1.025	0.575	1.828
30s	0.695	0.907	0.558	1.475	0.696	0.900	0.530	1.527
50s +	0.093	0.635	0.373	1.079	0.725	0.902	0.508	1.601
**Cohabitant (ref. no)**								
Yes	**0.013**	**0.557**	**0.351**	**0.886**	**0.007**	**0.498**	**0.302**	**0.823**
**Occupation (ref. doctor)**								
Nurse/nursing assistant	0.891	0.956	0.505	1.810	0.193	0.629	0.313	1.264
Pharmacist laboratory technician PT/ST/OT	0.764	0.898	0.444	1.815	0.405	0.721	0.333	1.558
Clerk/other	0.139	1.723	0.838	3.545	0.656	1.196	0.544	2.628
**Workplace (ref. ward)**								
Outpatient	0.801	1.079	0.599	1.944	0.657	1.154	0.613	2.172
Other	0.123	0.692	0.433	1.105	0.165	0.699	0.422	1.159
**Employment status (ref. full-time)**								
Part-time	0.293	0.681	0.332	1.395	0.617	0.821	0.380	1.776
**Night shift (ref. no)**								
Yes	0.317	1.259	0.802	1.978	0.626	1.130	0.692	1.843
**COVID-19 related work (ref. no/past)**								
Current	0.564	0.832	0.445	1.556	0.887	1.052	0.522	2.119
**Fear of COVID-19 and resilience**								
FCV-19S	**0.000**	**1.279**	**1.217**	**1.344**	**0.000**	**1.287**	**1.220**	**1.359**
RS14					**0.000**	**0.931**	**0.915**	**0.947**

The bold values indicate *p* < 0.05.

**FIGURE 1:**
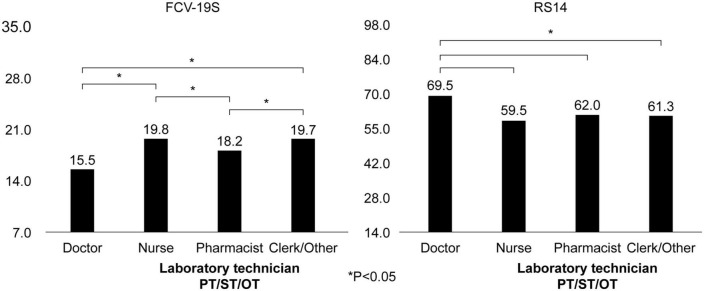
FCV-19S and RS14 in each occupation.

In a one-way analysis of variance to clarify the FCV-19S/RS14 for each job category, the FCV-19S was low for doctors (15.5 points) and high for nurses (19.8 points) and clerical workers/others (19.7 points); the RS14 was high for doctors (69.5 points) and low for nurses (59.5 points), pharmacists, laboratory technicians, physical therapists, speech therapists, or occupational therapists (62.0 points), and clerical workers/others (61.3 points) (Figure 1).

Regarding the association between FCV-19S and perceptions of hospital initiatives, it was found that being available for consultation regarding infection control in the hospital (*p* = 0.01, Cohen’s *d* = 0.26) and having access to psychological and emotional support (*p* = 0.04, Cohen’s *d* = 0.17) were associated with lower FCV-19S, but not with training on infectious diseases control or an adequate supply of personal protective equipment (Table 4).

**TABLE 4:** The associations between FCV-19S and perceptions of hospital initiatives.

	FCV-19S		
**Perceptions of hospital initiatives**	**Mean**	**P**	**Cohen d**
Training on infection control No (*n* = 352)	18.8	0.476	0.057
Yes (*n* = 282)	19.0		
Adequate supply of personal protective equipment No (*n* = 171)	19.3	0.171	0.123
Yes (*n* = 463)	18.7		
Available for consultation regarding infection control in the hospital No (*n* = 113)	**19.9**	**0.012**	**0.26**
Yes (*n* = 521)	**18.7**		
Available for psychological and emotional support in the hospital No (*n* = 247)	**19.4**	**0.035**	**0.172**
Yes (*n* = 387)	**18.6**		

The bold values indicate *p* < 0.05.

In summary, among HCWs during the COVID-19 pandemic, nurses and clerks experienced more mental distress than doctors. In contrast, HCWs in these occupations had stronger fear of COVID-19, as indicated by FCV-19S, and lower resilience, as indicated by RS14. Considering these psychological factors, there was no association between psychological distress and job title. In other words, psychological factors, such as fear of COVID-19 and resilience, played an important role in the mental health deterioration of nurses and clerks. Moreover, the availability of consultation regarding infection control at the hospital and psychological and emotional support services was important in reducing the fear of COVID-19.

## 4. Discussion

We conducted a survey on the mental health of HCWs during the COVID-19 pandemic to determine the relationship between factors such as fear of COVID-19 and resilience as well as mental distress.

It was found that females and younger participants seemed to experience more mental distress, however, no significant differences were found. Although many previous studies have reported that gender and age are associated with deteriorating mental health in HCWs during the COVID-19 pandemic (4, 10), a similar study of home-HCWs in Japan found no such association (14). It is unclear whether these associations differ from country to country or whether they are solely determined by the demographic characteristics of the population surveyed.

In this study, living with another individual was associated with mental distress only when FCV-19S was considered. While a previous study reported that living with another individual lowers the risk of mental health symptoms (34), and another study showed that HCWs feared infecting family members (35). The results of this study suggest that although living with someone may contribute to reduced mental health symptoms, a strong fear of transmission of COVID-19 may offset the benefits of living with others.

As in previous studies, mental health deteriorated among occupations other than doctors, such as nurses and clerks. According to previous studies, mental health has deteriorated in non-physician occupations in several countries (2, 4, 14). However, this association was lost in the present study, when FCV-19S was considered, while high FCV-19S levels were newly found to be associated with psychological distress. This suggests that fear of COVID-19 is a significant cause of psychological distress among nurses and clerical workers in hospitals treating patients with COVID-19. The higher total FCV-19S scores for other occupations compared to doctors also support this finding. In addition, the results are consistent with reports that the fear of COVID-19 has a negative impact on the mental health of HCWs (36) and that the FCV-19S is higher among nurses and clerical workers (14).

Moreover, resilience played an important role in explaining the association between job title and mental health. Logistic regression analysis showed that there was no significant difference between doctors and nurses when RS14 was considered, and the odds ratio for clerical workers was also lower. Additionally, resilience was lower in other occupations than in doctors. This result is consistent with previous reports, which show that resilience is a protective factor against pandemic stress among HCWs (37). The differences in mental health among occupations in this study may be partially explained by resilience. However, with regard to resilience by occupation, while there is evidence that doctors are highly resilient (38), as in this study, there is also evidence that doctors are less resilient than other occupations (39). The relationship between occupation in HCWs and their resilience may differ across countries, and further research is needed to examine it.

The results of this study suggest that mental health measures for HCWs during the COVID-19 pandemic may need to be implemented for a wide range of staff, including clerical staff, rather than targeting only those engaged in COVID-19-related work. Furthermore, the results suggest that efforts to reduce the fear of COVID-19 and to improve resilience may be effective. Although there are no definitive initiatives to reduce the fear of COVID-19, considering the factors associated with low FCV-19S, it seems important to build a support system that is not limited to one-way provision of knowledge, but is also interactive, such as providing emotional support and a point of contact for consultation regarding infection control, and making this information widely known. In addition, it was considered necessary to implement various interventions that have already been reported to improve resilience (40), and support individuals in obtaining enough rest, including sleep, and maintaining their quality of life during disasters (39).

The present study focused on psychological distress as measured by K6; however, in addition to general distress, Ide et al. (7) examined event-related distress and found that general and event-related distress were associated with isolation and exhaustion, while event-related distress was also associated with uncertainty. Moreover, fear of COVID-19 was associated with intolerance of uncertainty (41) and social isolation during the COVID-19 pandemic (42). Therefore, our results do not contradict those of the aforementioned studies; rather, given that severe general and event-related distress were a risk factor for suicidal ideation in Ide et al.’s study (7), the importance of fear of COVID-19 in the mental health of HCWs highlighted in the present study becomes more prominent.

However, there are certain limitations to the study. First, the exact number of staff at the time of the survey was unknown, and some staff members were hired, on leave of absence, or had retired during the survey. Moreover, the choice of the method of informing the staff about the survey was left open to each hospital. However, given that the size of each hospital covered in the study has not changed significantly in 2023, and based on the current number of staff, it can be estimated that there were approximately 7,000 staff members at the time of the survey. Therefore, the collection rate was about 10%, and given that it was a voluntary survey, the issue of representativeness is a limitation of this study. Second, it was not possible to determine causal relationships due to the cross-sectional nature of the survey. Third, owing to the small number of participants from professions other than doctors and nurses/nursing assistants, it was not possible to separately show the actual mental health status of those professions. Fourth, apart from fear of COVID-19 and resilience, there are several other factors, such as depression, anxiety, and stress that are related to the mental health of HCWs. In this study, we assessed psychological distress using the K6 as a mental health indicator. Thus, the study results represent only one aspect of the mental health status of HCWs during the COVID-19 pandemic.

## 5. Conclusion

This study found that the level of mental distress differed by occupation, but was not associated with COVID-19-related work, indicating that differences in the fear of COVID-19 and resilience were important. In order to provide mental healthcare to HCWs during a pandemic, it will be necessary to create consultation services where a wide range of employees can discuss their concerns and questions that arise during their work, rather than narrowing down intervention targets in advance. In addition, it was considered important to strengthen the resilience of HCWs in preparation for future disasters.

However, to counter the limitations of this study, there is a need to conduct studies with a larger sample size, longitudinal design, and that assess a variety of psychological factors. Furthermore, it is important to test the effectiveness of interventions for addressing the mental health issues of HCWs suggested in this study in future research.

## Data availability statement

The raw data supporting the conclusions of this article will be made available by the authors, without undue reservation.

## Ethics statement

This study involving human participants was reviewed and approved by the Medical Ethics Committee of the University of Tsukuba (No. 1546-3). The patients/participants provided their written informed consent to participate in this study.

## Author contributions

HT, IM, TA, and KY devised the project, the main conceptual ideas, and proof the outline. NK, KW, ST, YS, KN, SS, SD, and DH contributed to the data collection and preparation. HM analyzed the data and took the lead in writing the manuscript. HM and HT interpreted the results. All authors contributed to the article and approved the final version.
